# The Neglected Diseases Section in *PLoS Medicine*: Moving Beyond Tropical Infections

**DOI:** 10.1371/journal.pmed.0050059

**Published:** 2008-02-26

**Authors:** The PLoS Medicine Editors

## Abstract

The Neglected Diseases section will no longer focus on chronic tropical infections, but on other neglected health problems that have a major global burden.

Since our launch in October 2004, *PLoS Medicine* has published a policy and review section dedicated to neglected diseases. Its focus has been on the chronic tropical infections, mostly parasitic and bacterial, that burden the world's poorest people and that are also a major cause of global poverty. The many ways in which these diseases have been sidelined are well documented [1], and our main aim for the section was to help to place these conditions in the limelight. So what has the section achieved, and where is it heading next, particularly now that PLoS has launched a journal dedicated to the neglected tropical diseases (NTDs; http://www.plosntds.org/)?

The 21 articles published to date give a broad-ranging overview of the landscape of NTDs. Each article focused either on the global campaign to tackle a specific disease, such as trachoma or Buruli ulcer, or on a new strategy for approaching neglected diseases in general, including innovative ways of designing, developing, and funding antiparasitic drugs (see Text S1).

Many of these articles have challenged the widespread sense of hopelessness surrounding the NTDs. Mary Moran's analysis [2], for example, found that 63 neglected-disease drug projects were under way at the end of 2004, three-quarters of which are being conducted by public–private partnerships, often involving multinational or small-scale pharmaceutical firms. As we have written before, we are witnessing “a new era of hope for the world's most neglected diseases” [3].

The Neglected Diseases section has arguably had an impact upon the wider global health community, for example among policy makers and funding agencies. Two articles in particular stand out for their international influence. The first, by David Molyneux, Peter Hotez, and Alan Fenwick, presented the case for integrating the control of NTDs in Africa by scaling up a “rapid-impact package” of four antimicrobial drugs that have synergistic actions on multiple diseases [4]. This package, argued the authors, could tackle seven NTDs (schistosomiasis, trachoma, lymphatic filariasis, onchocerciasis, hookworm, trichuriasis, and ascariasis) at an annual cost of only about $US0.40 per person. The authors' follow-up article discussed the many opportunities for integrating NTD control with that of the “big three” diseases: HIV, malaria, and tuberculosis [5].

The first of these two articles led to a United Kingdom parliamentary question, by Member of Parliament Nicholas Soames, about the UK's funding of parasitic diseases [6], while the second helped make the case for a United Nations mandate to incorporate NTD control into its campaign to roll back malaria [7]. Both papers laid the foundation for the creation of a new global nonprofit organization, the Global Network for Neglected Tropical Disease Control (GNNTDC, http://gnntdc.sabin.org/). And the idea of integrated control, laid out in the two papers, has gained traction in the donor community—the United States Agency for International Development, for example, awarded a $US100 million grant to RTI International to scale up integrated control of NTDs in Africa [8], while Geneva Global Inc. awarded the GNNTDC US$8.9 million to deliver the “rapid-impact package” in Rwanda and Burundi [9].

*PLoS Medicine*'s Neglected Diseases section was also the catalyst for the launch of the world's first open-access journal specifically devoted to the NTDs. In 2005, the section caught the attention of Professor Peter Hotez at George Washington University and the Sabin Vaccine Institute, who proposed to PLoS that we launch *PLoS Neglected Tropical Diseases*. One of us (GY) presented the idea at the World Health Organization's (WHO's) “Strategic and technical meeting on intensified control of neglected tropical diseases” in Berlin in April 2005 [10], which led to the WHO's Department of Neglected Tropical Diseases giving the proposal its formal support. The momentum continued to build—several global leaders in NTDs agreed to serve on the journal's editorial board, and the Bill & Melinda Gates Foundation awarded PLoS a $US1.1 million grant to cover the journal's launch phase.

*PLoS Neglected Tropical Diseases*—which publishes research, commentary, analysis, and reviews on the pathology, epidemiology, treatment, control, and prevention of the NTDs—launched at the end of October 2007. Its arrival was heralded in the *New York Times* (November 6, 2007) with the headline “Shining Light on Diseases Often in the Shadows” [11], while an editorial in *The Lancet* about the new journal praised the “investment in scaling up communication about global health” [12]. The journal publishes the broadest range of NTDs research in a single open-access venue, while its Magazine section advocates for those who suffer from the plight of these tropical infections. Built upon an online publishing platform called Topaz (see http://www.plos.org/cms/node/36/), the journal takes advantage of the latest “Web 2.0” tools from PLoS, including readers' annotations, discussion threads, and article rankings. Its editorial board is well represented by developing-world scientists (http://www.plosntds.org/static/edboard.action).

The arrival of PLoS' latest journal means that it now makes little sense for the *PLoS Medicine* Neglected Diseases section to focus on the NTDs. This month's article, examining the WHO strategy to eliminate human African trypanosomiasis, therefore marks the last time that this section will feature a tropical infection [13]. Instead, the section will focus on other health problems that could be considered neglected and that have a significant global burden. We will ask authors to justify in their article, with supporting evidence, why they consider the problem to be neglected, to outline the scale of the problem worldwide, and to discuss possible policies and solutions for reducing the burden of disease. Examples might include reproductive and maternal health problems, mental illness in low- and middle-income countries, road traffic injuries, and health problems related to migration and conflict. Authors should always send a pre-submission inquiry via our online journal management system (http://medicine.plosjms.org/cgi-bin/main.plex) so that we can assess the suitability of the topic ahead of formal submission.

PLoS continues to receive requests to launch new journals. In this issue of *PLoS Medicine*, for example, Basia Zaba and colleagues make the case for a new open-access journal specializing in research on demographic surveillance—the process of monitoring births, deaths, causes of death, and migration in a population over time [14]. Our response to these requests is this: *PLoS ONE* (http://www.plosone.org/) publishes *any* study in science and medicine that is technically, scientifically, and ethically sound, subject to peer review, so there is now a home at PLoS for *all* original biomedical research. Indeed *PLoS ONE* has already published research based on demographic health surveys, such as a study that used the 1998 South African Demographic and Health Survey to examine the association between measures of socioeconomic position and violence at the individual and household levels [15].

As PLoS continues to evolve, we plan to create “hubs” of linked open-access content from across the PLoS journals to help serve specific research communities (see http://www.plos.org/about/faq.html#ploshubs); our first such hub is the PLoS Hub for Clinical Trials at http://clinicaltrials.ploshubs.org/. We hope that these innovative new ways of presenting scientific research, from all corners of the globe and on topics that have traditionally been sidelined by subscription-based journals, will help take the “neglected” out of the term “neglected diseases.”

## Supporting Information

Text S1 Articles published in the Neglected Diseases section from October 2004–February 2008, focusing on tropical infections (30 KB DOC).

## Abbreviations

GNNTDC: Global Network for Neglected Tropical Disease Control
NTD: neglected tropical disease
WHO: World Health Organization
