# Supplementary material for: The Neglected Diseases Section in PLoS Medicine: Moving Beyond Tropical Infections
Source: PLoS Med. 2008 Feb 26;5(2):e59. doi: 10.1371/journal.pmed.0050059 (PMC2253615; doi:10.1371/journal.pmed.0050059)
Supplement: Text S1 — (30 KB DOC). [file pmed.0050059.sd001.doc]

**Supporting Information Text S1**

Articles published in the Neglected Diseases Section from October 2004-February 2008, focusing on tropical infections.

**Specific diseases**

- Blinding Trachoma: A Disease of Poverty (PLoS Med 1(2): e44)
- Coccidioidomycosis—A Fungal Disease of the Americas (PLoS Med 2(1): e2)
- Hookworm: “The Great Infection of Mankind” (PLoS Med 2(3): e67)
- Buruli Ulcer (M. ulcerans Infection): New Insights, New Hope for Disease Control (PLoS Med 2(4): e108)
- Visceral Leishmaniasis: New Health Tools Are Needed (PLoS Med 2(7): e211)
- The Global Campaign to Eliminate Leprosy (PLoS Med 2(12): e341)
- Confronting the Neglected Problem of Snake Bite Envenoming: The Need for a Global Partnership (PLoS Med 3(6): e150)
- River Blindness: A Success Story under Threat? (PLoS Med 3(9): e371)
- Liver Fluke Induces Cholangiocarcinoma (PLoS Med 4(7): e201)
- The Challenges of Chagas Disease— Grim Outlook or Glimmer of Hope? (PLoS Med 4(12): e332)
- [Cytomegalovirus Retinitis: The Neglected Disease of the AIDS Pandemic](http://medicine.plosjournals.org/perlserv/?request=get-document&doi=10.1371/journal.pmed.0040334) (PLoS Med 4(12): e334)
- Plague: Past, Present, and Future (PLoS Med 5(1): e3)
- Human African Trypanosomiasis Elimination: Where Do We Stand and What Comes Next? (PLoS Med 5(2): e55)

**New strategies for NTD control**

- New Drugs for Neglected Diseases: From Pipeline to Patients (PLoS Med 1(1): e6)
- Finding Cures for Tropical Diseases: Is Open Source an Answer? (PLoS Med 1(3): e56)
- The Courage to Change the Rules: A Proposal for an Essential Health R&D Treaty (PLoS Med 2(2): e14)
- Designing Drugs for Parasitic Diseases of the Developing World (PLoS Med 2(8): e210)
- A Breakthrough in R&D for Neglected Diseases: New Ways to Get the Drugs We Need (PLoS Med 2(9): e302)
- “Rapid-Impact Interventions”: How a Policy of Integrated Control for Africa’s Neglected Tropical Diseases Could Benefit the Poor (PLoS Med 2(11): e336)
- Incorporating a Rapid-Impact Package for Neglected Tropical Diseases with Programs for HIV/AIDS, Tuberculosis, and Malaria (PLoS Med 3(5): e102)
- Anaemia: A Useful Indicator of Neglected Disease Burden and Control (PLoS Med 4(8): e231)
